# Continuous Spectrophotometric Assay for Defluorinase and Dechlorinase Activities With α‐Halocarboxylic Acids

**DOI:** 10.1111/1751-7915.70216

**Published:** 2025-08-13

**Authors:** Marie Ronnander, Anthony G. Dodge, Erin O'Neal, Caroline Pauls, Jack Hanson, James K. Christenson, Lawrence P. Wackett

**Affiliations:** ^1^ Department of Biochemistry, Molecular Biology and Biophysics and Biotechnology Institute University of Minnesota St. Paul Minnesota USA; ^2^ Department of Chemistry Bethel University St. Paul Minnesota USA

**Keywords:** activity determination, bacteria, coupled, dehalogenase, dehydrogenase, enzyme, organofluorine, PFAS

## Abstract

Many environmental pollutants have a fluorine or chlorine atom on a carbon atom adjacent to a carboxylic acid. These α‐halocarboxylic acids include heavily regulated compounds such as per‐ and polyfluorinated substances (PFAS). Due to PFAS persistence in the environment, there is intense interest in characterising the biodegradation of α‐halocarboxylic acids. Their initial biodegradation often proceeds via defluorinase enzymes that catalyse hydrolytic removal of *alpha* fluorine or chlorine atoms. These enzymes can dehalogenate both mono‐halocarboxylate and dihalocarboxylate substrates, generating α‐hydroxy and α‐ketocarboxylic acid products, respectively. To enable continuous monitoring of defluorinase activity, we identified, purified and optimised dehydrogenases from *Limosilactobacillus fermentum* JN248 and *Enterococcus faecium* IAM10071 that reacted with the specific α‐hydroxy and α‐ketocarboxylic acid products of the defluorinases. The dehydrogenases make or consume NADH, measured by absorbance readings at 340 nm, thus allowing continuous measurement of defluorinase activity using a spectrophotometer. Using the coupled assay, purified defluorinases from a *Delftia* sp. and a *Dechloromonas* sp. were compared with respect to substrate specificity. The *Delftia* defluorinase demonstrated superior activity with most substrates, including difluoroacetate. To our knowledge, this is the first report of a coupled‐enzyme continuous assay method for enzymes that catalyse the hydrolysis of α‐halocarboxylic acids.

## Graphical abstract

Microbial hydrolytic defluorinases and dechlorinases assayed by coupling the reaction product to NAD‐linked dehydrogenases will facilitate screening for and characterising new and known enzymes.

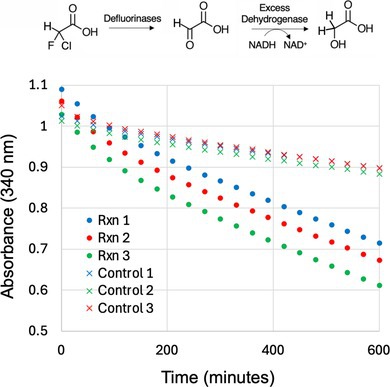

## 1 Introduction

**FIGURE 1:**
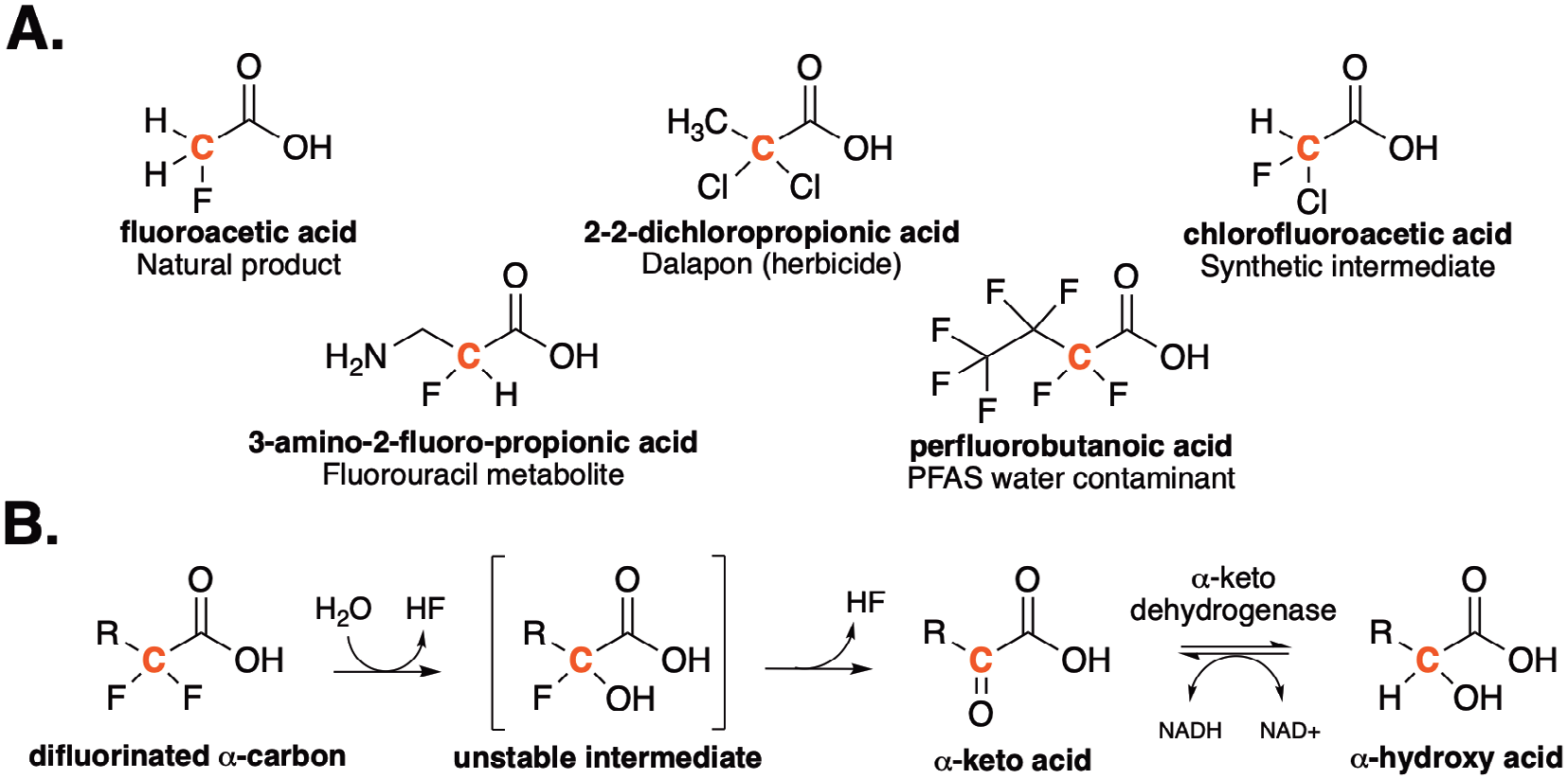
Important alpha‐halocarboxylic acids, their biodegradation pathway and concept of the coupled assay. (A) Common α‐ and α, α‐halocarboxylic acids used today, and (B) the breakdown process of α, α‐halocarboxylic acids, which form substrates for dehydrogenase enzymes. The red carbon atom in the figure highlights the alpha‐ (α‐) position.

Halogenated compounds have many commercial applications and constitute an important class of environmental pollutants. Chlorinated compounds were heavily used in the mid‐twentieth century, while per‐ and polyfluorinated alkyl substances (PFAS) have become prominent more recently (Wackett and Robinson 2020; Schymanski et al. 2023). Figure 1A shows the fluorinated natural product fluoroacetic acid and more complex halogenated commercial compounds or metabolites. Dalapon was a widely used chlorinated herbicide (Marchesi and Weightman 2003); chlorofluoroacetic acid is used as a synthetic intermediate (Meyer et al. 2019); 3‐amino‐2‐fluoropropionic acid is a metabolite derived from the cancer drug fluorouracil (Johnson et al. 2020); and perfluorobutanoic acid (PFBA) is a widespread PFAS environmental contaminant (Liang et al. 2023). PFBA is representative of the most heavily regulated PFAS class of compounds that includes perfluorooctanoic acid (PFOA) (Glüge et al. 2020; Evich et al. 2022). There is widespread interest in tracking, and ultimately degrading, these chemicals. In this study, dechlorinase and defluorinase enzymes are sought to detoxify and degrade these halogenated pollutants.

Fluoroacetic acid and many PFAS compounds of concern have a fluorine substituent *alpha* to a carboxylic acid group (Figure 1A,B) (Chan et al. 2011; Probst et al. 2025). The enzymes that displace halide substituents, including fluorine, from the α‐carbon belong to two major enzyme superfamilies. Fluoroacetate dehalogenases have an α/β hydrolase fold and likely evolved to react with the natural product fluoroacetic acid (Chan et al. 2011). These enzymes are also observed to displace chlorine substituents. Another distinct enzyme family, the haloacid dehalogenases, also contains member enzymes that catalyse the displacement of chlorine or fluorine from carbon atoms *alpha* to a carboxylic acid group (Chan et al. 2022; Probst et al. 2025).

Both enzyme classes catalyse hydrolytic dehalogenation reactions. When they react with substrates that contain one halogen substituent on the α‐carbon, the product is an alcohol and a hydrogen halide. A few of the enzymes in both enzyme families have been shown to also act on the two fluorine atoms of difluoroacetate (Khusnutdinova et al. 2023; Probst et al. 2025). When the substrate has an α, α‐difluoro carbon atom, the replacement of one fluorine atom with a hydroxyl group produces a *gem*‐fluoro alcohol (Figure 1B). *Gem*‐fluoro alcohols are known to undergo a rapid, spontaneous elimination of HF to generate a ketone (Seppelt 1977). Thus, α‐hydroxy or α‐ketocarboxylic acids are the two possible products.

Both α‐hydroxy and α‐ketocarboxylic acids are known to undergo interconversion by dehydrogenase enzymes that are ubiquitous in prokaryotes, plants and animals, with lactate dehydrogenase being a well‐known example. These enzymes commonly use NADH as an electron donor and NAD^+^ as an electron acceptor. The reactions are typically reversible. Since the dehalogenases produce alcohols or ketones as products, NAD(H)‐linked dehydrogenases may be used to measure either product. The reaction with alcohols will produce NADH; the reaction with ketones will consume NADH.

The consumption or formation of NADH or NAD^+^, respectively, can be measured at 340 nm (A_340_) using a spectrophotometer. In this present study, we aimed to develop a continuous colourimetric assay for defluorinase enzymes by coupling defluorination products to the formation or consumption of NADH using dehydrogenases with broad specificity. Future studies may use this assay method not only with α‐fluorocarboxylic acids, but also generally with α‐halocarboxylic acids.

## 2 Experimental Procedures

### 2.1 Chemicals

The chemicals used were obtained from the vendors indicated: kanamycin sulfate, HEPES, isopropyl‐beta‐D‐thiogalactoside (IPTG) (GoldBio, Saint Louis, MO, USA); Tris base, hydrochloric acid, sodium chloride, glyoxylic acid, glacial acetic acid (ThermoFisher Scientific, Rockford, IL, USA); sodium phosphate monobasic, NAD^+^, NADH disodium salt, D‐mandelic acid (MilliporeSigma, Saint Louis, MO, USA); dichloroacetic acid, D‐lactic acid, 2,2‐difluoropropionic acid, chloroacetic acid, difluoroacetic acid (Oakwood Chemical, Estill, SC, USA); (*RS*)‐α‐fluorophenyl acetic acid (Enamine, Kyiv, Ukraine); chlorofluoroacetic acid, (*RS*)‐α‐fluoropropionic acid, bromofluoroacetic acid (AmBeed, Buffalo Grove, IL, USA); 2‐chloro‐2,3,3,3,3‐tetrafluoropropionic acid, trifluoropyruvic acid (A2B Chem, San Diego, CA, USA).

### 2.2 Growth Media and Conditions

All strains were grown on platform shakers (200 rpm) in Difco Miller Lysogeny Broth (LB) (Becton‐Dickinson, Sparks, MD, USA) or on LB solidified with 1.5% Difco Bacteriological agar (Becton‐Dickinson) in Petri plates. *Escherichia coli* strains were grown at 37°C or as indicated, and *Pseudomonas putida* ATCC 12,633 strains were grown at 28°C. All media for recombinant strains contained 50 μg/mL kanamycin sulfate for plasmid maintenance or selection.

### 2.3 Gene Synthesis and Cloning

A table for strains and genes for this study can be found in the Supporting Information (Table S1). Two defluorinases were used from previous studies: *Delftia acidovorans* strain B (GenBank accession BAC81979) (previously *Mooroxella* sp. strain B, Liu et al. 1998) (referred to here as DEF1) and Dechloromonas aromatica RCB (PDB ID 8SDC), Khusnutdinova et al. 2023 (referred to here as DEF2). Both were previously codon‐optimised, synthesised and inserted into expression vectors as described by Dodge et al. (2024) and O'Connor et al. (2024), respectively. Two dehydrogenases were selected: a lactate dehydrogenase (LDH) from Limosilactobacillus (formerly Lactobacillus) fermentum JN248 (Chen et al. 2017) and a D‐mandelate dehydrogenase (MDH) (also known as 2‐ketopantoate reductase, D‐2‐hydroxyacid dehydrogenase or 2‐dehydropantoate 2‐reductase) from *Enterococcus faecium* IAM10071 (Wada et al. 2008). Both dehydrogenases were codon‐optimised for expression in *E. coli* BL21(DE3) using the Integrated DNA Technologies (IDT, Coralville, IA, USA) codon optimisation web tool and were synthesised by IDT with codons encoding six histidine residues and a thrombin cleavage site added to the 5′ ends. Codon‐optimised dehydrogenase sequences are reported in supporting information (Figure S1). A New England BioLabs (NEB, Ipswitch, MA, USA) HiFi DNA Assembly Cloning Kit was used to insert the synthetic fragments into pET‐28b+ (MilliporSigma) plasmids that were linearised by digestion with NcoI and XhoI (NEB). The assembly reactions were used to transform high‐efficiency *E. coli* NEB 5‐alpha cells, and transformants were selected on LB agar plates with kanamycin. Plasmids were isolated from transformants using a QIAprep spin mini‐prep kit (QIAGEN, Hilden, Germany) and complete sequences were confirmed by nanopore sequencing (Plasmidsaurus, Eugene, OR, USA). Expression strains were created by transforming *E. coli* BL21(DE3) (NEB) with the verified plasmids.

### 2.4 Enzyme Expression and Purification

All proteins were purified via immobilised‐metal affinity chromatography (IMAC). DEF1 was expressed constitutively from a T5 promoter in *P. putida* ATCC 12,633 as described previously (Dodge et al. 2024); all other proteins were expressed from a T7 promoter by induction with 1 mM IPTG. Inocula (10 mL) were started from single colonies picked from plates and grown overnight. The entire overnight cultures were transferred into 1 L aliquots of media in 4 L Erlenmeyer flasks and incubated with shaking until OD600 nm reached 0.4–0.7 as read in 1 cm pathlength polystyrene cuvettes (Sarstedt, Nümbrecht, Germany) with a Cary 60 UV–vis spectrophotometer (Agilent, Santa Clara, CA, USA). The cultures were cooled in a room temperature water bath for 15 min and were then transferred to a shaking platform at 16°C to cool for another 45 min before the IPTG was added. Incubation with shaking at 16°C was continued for another 18 h. The cells were then harvested by centrifugation at 6000 × *g* and resuspended in 15 mL of 50 mM Tris‐Cl (pH 7.5) with 200 mM NaCl (defluorinases) or 20 mM NaPO4 (pH 7.4) with 500 mM NaCl (dehydrogenases). A Pierce EDTA‐Free Mini Protease Inhibitor tablet (ThermoFisher) was added to the cell suspensions, and the cells were lysed using a French pressure cell (3 cycles at 140 MPa). The resulting crude lysates were centrifuged at 19000 × *g* for 90 min, and the supernatants (cleared lysates) were injected onto a HisTrap FF column (Cytiva, Marlborough, MA, USA) charged with Ni^2+^ that was installed on a GE Healthcare (Cytiva) ÄKTA fast liquid protein chromatography (FPLC) system. Isocratic washes and gradient elutions were conducted with the buffers above, paired with their analogues containing 500 mM imidazole. Low‐affinity and weakly bound proteins were then eluted with washes of 10 or 50 mM imidazole, respectively. His‐tagged proteins bound to the column were then eluted with a linear gradient from 50–250 mM imidazole while collecting 3 mL fractions. Fraction purity was checked by SDS‐PAGE in 12.5% gels, fractions of equal purity were pooled and then the proteins were concentrated and the buffer was exchanged with 20 mM HEPES (pH 7.5) + 200 mM NaCl using Amicon Ultra 10 kDa MWCO centrifugal concentrators (MilliporeSigma). Aliquots of the proteins in microcentrifuge tubes were flash frozen in liquid nitrogen and then stored at −80°C.

### 2.5 Measuring Defluorinase Activity With an Ion‐Specific Electrode

Stock solutions (100 mM) of racemic mono‐fluorinated substrates (containing 50 mM of the reactive (S)‐enantiomers (Wang et al. 2017; Dodge et al. 2024)) or di‐ or multi‐fluorinated substrates (non‐chiral) were prepared in 0.1 M Tris base with pH adjusted to 9.0 or 8.0, respectively, with NaOH or HCl as needed. Triplicate 0.5 mL reactions containing 30 mM halogenated compound in 0.1 M Tris (pH 9.0 or 8.0 as above) were incubated with 2–400 μg of purified defluorinase in 15 × 85 mm borosilicate glass tubes (ThermoFisher) at room temperature. Reactions were stopped by the addition of 0.5 mL of total ionic strength adjustment buffer (TISAB: 1 M glacial acetic acid, 1 M NaCl, 10 mM 1,2‐cyclohexanedinitrilotetraacetic acid (CDTA), pH 5), and then, free fluoride ion was measured with a Fluoride Ionplus Sure‐Flow Combination ISE fluoride electrode that was connected to an Orion Star A214 pH/ISE meter (ThermoFisher). Control incubations of each compound without enzyme added were also done to check for spontaneous fluoride release.

### 2.6 Determining the pH Optima of DEF1 and DEF2

The defluorination activity of DEF1 of DEF1 from pH 6.0–11.0 was measured in triplicate 0.5 mL reactions containing 2 μg enzyme and 20 mM α‐fluorophenylacetic acid (α‐FPhAA) in 0.1 M MES (pH 6.0–6.5), HEPES (pH 6.5–8.0), bicine (8.0–9.0), CHES (9.0–10.0) or CAPS (pH 10.0–11.0). Reactions were incubated for 20 min at room temperature and then analysed for free fluoride as above. The pH curve for DEF1 was repeated later with 30 mM α‐FPhAA in 0.1 M Tris (pH 7.0–9.5) at 30°C.

### 2.7 Measuring Dehydrogenase Activity

Activity of the purified dehydrogenases and purchased rabbit heart lactate dehydrogenase (MilliporeSigma) was determined via continuous assays in which either reduction of NAD^+^ (increase of A_340_) or oxidation of NADH (decrease of A_340_) was tracked in 1 mL reactions in quartz cuvettes using a Cary 60 spectrophotometer for 5 min. Reactions contained 0.10–5.0 μg of dehydrogenase plus 30 mM α‐hydroxycarboxylic acid (D‐lactate or D‐mandelate) and 25 mM NAD^+^ in 0.1 M Tris (pH 9.0); or 30 mM α‐ketocarboxylic acid (glyoxylate, pyruvate, trifluoropyruvate or benzyoyl formate) and 0.300 mM NADH in 0.1 M Tris (pH 9.0). Activity was measured as the maximum linear ΔA_340_ min^−1^. Because the reduction of an α‐ketocarboxylic acid coupled to NADH oxidation by a dehydrogenase is more favourable than the oxidation of an α‐hydroxycarboxylic acid coupled to NAD^+^ reduction, the linear portion of the NADH formation curve was typically limited to about 1 min due to the unfavourable equilibrium leading to reversibility of the reaction. Unless otherwise noted, final activity data are the average of three technical replicates of the same enzyme purification batch and are reported with two significant figures to best reflect the precision of the measurements.

### 2.8 Coupled Dehalogenation Assays Using Defluorinases and Dehydrogenases

Triplicate coupled reactions (1 mL) containing a defluorinase and a dehydrogenase were conducted using the buffering conditions, halogenated substrate concentrations and the NAD^+^ or NADH concentrations described above. Dehalogenation was inferred by tracking ΔA_340_ resulting from the coupling of NAD^+^ reduction or NADH oxidation to the oxidation or reduction of the α‐hydroxycarboxylic acid or α‐ketocarboxylic acid product of the defluorinase by the dehydrogenase. Reaction monitoring and activity determinations were as above for the dehydrogenase assays. Reactions with di‐ or multi‐halogenated acids (produce α‐ketocarboxylic acid products and consume NADH) typically contained 20 μg DEF1 or DEF2 and 200 μg LDH or MDH. Reactions with mono‐halogenated acids (produce α‐hydroxycarboxylic acid products and form NADH) contained 1.0 μg DEF1 or DEF2 and 50 μg LDH or MDH. Coupled assays were also run in 96‐well microtiter plates under similar conditions using a SpectraMax iD3 plate reader (Molecular Devices, San Jose, CA, USA). Reaction volumes were decreased to 200 μL, and the NADH concentration was increased to 400 μM. For all coupled assay formats, the most commonly added amounts of defluorinase are listed above, but the amounts varied from 0.001 to 40 μg to accurately assay very fast or very slow dehalogenation of specific substrates. All reactions were calibrated so that the units of dehydrogenase added were at least 100× greater than the units of defluorinase added for the specific substrate being measured. Lower and upper detection limits were defined by rejecting slopes < 0.0020 or > 0.3 Abs/min.

### 2.9 Identifying the Organic Defluorination Product of Difluoroacetic Acid by HPLC

Reactions (1 mL) contained 30 mM DFAA in 0.1 M Tris (pH 8.5). Negative controls used an equivalent volume of the HEPES/NaCl enzyme storage buffer in place of DEF1. After incubation at room temperature for 20 h, 0.5 mL of each reaction or control was transferred into Amicon Ultra 0.5 mL centrifugal filters (10,000 MWCO) (MilliporeSigma) and centrifuged for 15 min at 16,900 × *g*. The deproteinised filtrates (flow‐through) were transferred into 2 mL glass vials that were then sealed with PTFE/silicone‐lined screw caps (Chrom Tech, Apple Valley, MN, USA). Filtrate aliquots (10 μL) were injected onto a Thermo‐Fisher Acclaim Mixed‐Mode WAX‐1 column (4.6 × 150 mm, 5 μm particle size) using a Hewlett‐Packard (now Agilent) 1100 series high‐pressure liquid chromatography (HPLC) system. Elution was done with an isocratic mobile phase (25 mM phosphate buffer, pH 6.0) at a flow rate of 0.8 mL/min, and chromatograms were acquired by monitoring at 210 nm with a diode array (UV–vis) detector. Peak identities were assigned by comparing observed peak retention times with the retention times of commercial standards acquired under the same conditions. Relative quantitation was done by comparing peak areas that were acquired by manual integration of each peak.

### 2.10 Using the Coupled Assay to Measure Reaction Kinetics of DEF2 With Chlorofluoracetic Acid

Data to derive Michaelis–Menten kinetic parameters were acquired from coupled assays that were conducted in 96‐well plates as described above with 40 μg DEF2, 50 μg LDH, 400 μM NADH and chlorofluoroacetic acid at concentrations from 1 to 30 mM. Activity was determined as the absolute value of the slope of ΔA_340_ from 1 to 5 min. A reaction with 1.0 mM chlorofluoroacetic acid (CFAA) and no DEF2 was used as the negative control; the slope was subtracted from each slope measured with the substrate present. Data were fit to the Michaelis–Menten equation using Mathematica (Version 14.2, Wolfram Research Inc., Champaign, IL) and *k*_cat_ and *K*_m_ were calculated.

## 3 Results

### 3.1 Selection, Purification and Initial Activity Screening of Defluorinases

Previously characterised defluorinases (DEF1 and DEF2) (Liu et al. 1998; Khusnutdinova et al. 2023; Dodge et al. 2024) were reported to be reactive with aliphatic and aromatic α‐substituted mono‐ and difluorinated carboxylic acids were chosen for this study. Both enzymes react only with (*S*)‐monofluoro‐enantiomers to produce alcohols (α‐hydroxycarboxylic acids) with the (*R*)‐configuration (Wang et al. 2017; Dodge et al. 2024). Ketones (α‐ketocarboxylic acids) are produced from the achiral α, α‐difluorosubstrates.

Both defluorinases were purified to > 90% purity (Figure S2) in a single step as described in Materials and Methods. Total yields were 86 or 134 mg of purified DEF1 or DEF2, respectively, from 1 L of culture. Specific activity of the purified defluorinases was determined by detecting liberated fluoride ion when reacted with α‐fluorophenylacetic acid (α‐FPhAA), and the results for DEF1 (14 ± 1 μmol min^−1^ mg^−1^) or DEF2 (33 ± 1 μmol min^−1^ mg^−1^) were consistent with previous determinations (Dodge et al. 2024; O'Connor et al. 2024). Activities of both defluorinases on α‐FPhAA were determined in a set of Good's buffers (Tris not included) over a pH range from 6.0 to 11.0, and a broad optimum from pH 8.0 to 9.0 was observed for both enzymes (Figure S3). This was repeated with DEF1 from pH 7.0 to 9.5 using only Tris buffer and showed maximum activity at pH 8.5–9.0, a slight decline at pH 9.5 (89% of max) and a steeper drop at pH 8.0 (75% of max). The optimum for DEF1 was previously reported as pH 9.5 (Liu et al. 1998); a pH optimum for DEF2 had not been reported previously.

During initial experiments, we unexpectedly observed that the activity of DEF1 with difluoroacetic acid (DFAA) in Tris buffer was over 3× higher than the activity measured at the same pH in HEPES, Bicine, Tricine or phosphate buffers (Table S2). This buffer effect was not observed with any other substrate nor with DEF2. A mechanistic explanation of this buffer effect is currently unknown, but all subsequent reactions were conducted in Tris.

### 3.2 Selection, Purification and Initial Activity Screening of Dehydrogenases

Dehydrogenases (DH) were required to couple the diverse α‐ketocarboxylic acid and α‐hydroxycarboxylic acid dehalogenation products to NADH formation or consumption visible at A_340_. Initially, we purchased rabbit heart lactate dehydrogenase because earlier reports stated it was reactive with both lactate (C_3_) and glyoxylate (C_2_) (Banner and Rosalki 1967). Although we could couple NADH oxidation to glyoxylate produced by the dehalogenation of chlorofluoroacetate (CFAA), the activity with commercial rabbit heart lactate dehydrogenase with glyoxylate was too slow to make scaling the assay practical. Subsequently, we selected a lactate dehydrogenase (LDH) from *Limosilactobacillus fermentum* JN248 and a mandelate dehydrogenase (MDH) from *Enterococcus faecium* IAM10071 that were reported in the literature to have high activities and broad specificities (Chen et al. 2017; Wada et al. 2008).

**FIGURE 2:**
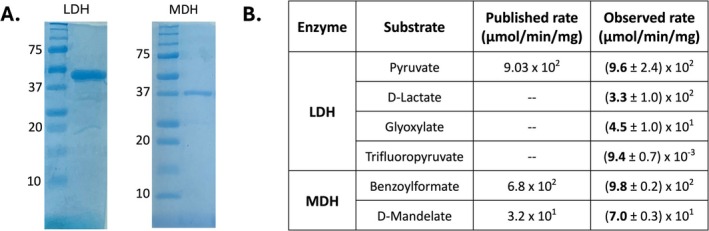
Coupling enzymes, purification and reaction rates with different substrates. (A) SDS‐PAGE of purified lactate dehydrogenase from *L. fermentum* (LDH) and mandelate dehydrogenase from *E. faecium* (MDH). The column of numbers adjacent to the gels indicates the MW (kDa) of the corresponding protein size standards. (B) Dehydrogenase specific activity previously reported (Published rate) or measured in this study (Observed rate).

Both dehydrogenations were purified in a single step as described in Materials and Methods with yields of 68 or 185 mg of purified protein per litre of induced culture for LDH or MDH, respectively (Figure 2A). Both LDH and MDH purified well and demonstrated good activity against various substrates we intended to assay (Figure 2B). LDH and MDH activities aligned with the literature except for mandelate oxidation, for which we observed > 20× higher activity than what was reported. Of note, LDH showed low but detectable activity with the perhalogenated substrate, trifluoropyruvate (TFPA) (Figure 2B), a product that would be expected from the α‐defluorination of perfluoropropionic acid. It was not obvious that TFPA would be reduced by LDH as the compound exists largely or entirely as the dihydrate, CF_3_C(OH)_2_COOH and some lactate dehydrogenases are unreactive with this substrate (Pogolotti Jr and Rupley 1973). For the remainder of the work, LDH was used exclusively for C_2_ and C_3_ aliphatic carboxylic substrates, and MDH was used for aromatic carboxylic acid substrates.

### 3.3 Coupled Assay Optimisation for Mono‐ and Dihalo‐Substrates

**FIGURE 3:**
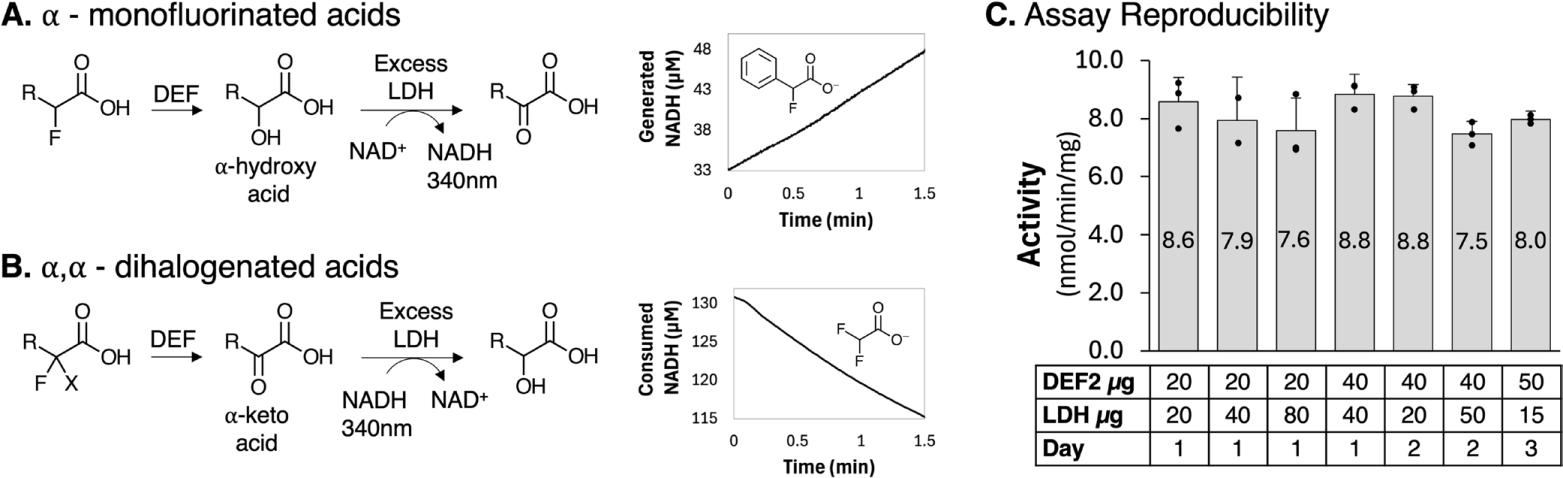
Characterising the coupled assay for mono‐ and dihalo‐substrates. (A) Schematic of coupled reactions for substrates where *R* = F, Cl, Br, CH_3_, CF_3_, phenyl, where X = H, F, CH_3_, and where DEF represents a defluorinase. (B) Representative reaction traces for both NAD+ reduction and NADH oxidation. (C) Reproducibility of the couple assay with *Dechloromonas aromatica* as the source of the defluorinase (DEF2), *Limosilactobacillus fermentum* as the source of the lactate dehydrogenase (LDH), and chlorofluoroacetate as the substrate. The second experimental set on Day 1 contains only two data points.

The coupled assays were optimised for both single determinations in cuvettes and for multiple simultaneous determinations in 96‐well microtiter plates, as would be practical for high‐throughput screening. We coupled dehydrogenase and dehalogenase reactions in both directions—NADH formation or consumption (Figure 3A,B). In the direction of NADH formation from α‐hydroxycarboxylic acid oxidation, the equilibrium is unfavourable, and the reversibility of the reaction limits the period in which a linear increase in A_340 nm_ could be observed (15–60 s). In the direction of NADH consumption from α‐ketoacid reduction, the equilibrium is favourable and reactions proceed at constant rates to near completion. The specific compositions of the coupled assay reactions are described in Materials and Methods.

The reproducibility of this coupled assay was tested with repeated reactions of DEF2 with CFAA, and the activity was found to be reproducible over multiple days and with various amounts of DEF2 and LDH included (Figure 3C). To accurately determine activity, the rate‐limiting step of the coupled reactions must be dehalogenation of the substrate by the defluorinase and not oxidation/reduction of the dehalogenated product by the dehydrogenase. To achieve this, we always ensured the specific activity of the dehydrogenase was over 100× the measured specific activity of the dehalogenase. This standard was adopted for all subsequent assays.

### 3.4 Validating the Coupled Assay by Comparison With Direct Fluoride Detection

We tested the efficacy of the coupled assay vs. fluoride detection with an ion‐specific electrode using known substrates for defluorinases: DFAA, α‐fluoropropionic acid (α‐FPA), CFAA and α‐FPhAA. However, the mechanism of DFAA defluorination was unclear in the literature. Khusnutdinova et al. (2023) proposed that defluorinases displace both fluorine atoms consecutively; whereas another study suggested that a single hydrolytic defluorination can generate a *gem*‐fluoro alcohol that rapidly and spontaneously eliminates HF (Aukema et al. 2022). Since our assay depends on the presence of an alcohol (α‐hydroxycarboxylic acid) or ketone (α‐ketocarboxylic acid), we first used the LDH‐coupled assay and HPLC to confirm the product. A rapid decrease in A_340_ was observed when LDH and NADH were added to deproteinised overnight reactions of DEF1 with DFAA, indicating the presence of an α‐ketocarboxylic acid. HPLC analysis revealed the appearance of a large peak with the same retention time as a commercial glyoxylate standard that was absent in the control without DEF1 (Figure S4). Additionally, the DFAA peak area declined by 82% in the presence of DEF1, and a corresponding amount of free fluoride was detected using the fluoride electrode (Figure S4). All experiments showed glyoxylate as the product of DFAA with DEF1. We did not test DEF2.

After establishing the glyoxylate product of DFAA, we compared the activity of DEF1 or DEF2 with four known substrates as determined by the dehydrogenase coupled assay or with the fluoride electrode (Table 1). The results showed that both methods could measure activity over nearly five orders of magnitude (0.0026 for DFAA to 33 for α‐FPhAA with DEF2). Both methods produced surprisingly similar results on the same substrates, with only the activity of DEF1 with CFAA or DEF2 with α‐FPA differing by ≥ 3× between the two methods (Table 1). In both cases, the higher activity was measured with the coupled assay. This effect was not observed with all substrates and may have been coincidental, but one advantage of continuous assays is the ability to measure initial rates more accurately. Reactions for the fixed timepoint assay using the fluoride electrode were incubated longer and were theoretically more susceptible to product inhibition or enzyme inactivation, but this was not investigated here. DEF1 had not previously been reported to react with DFAA, and, surprisingly, its activity was greater than one order of magnitude higher than DEF2 with both the fluoride electrode and coupled enzyme assays.

**TABLE 1:** Specific activities (*μmol min* ^*−1*^ *mg* ^*−1*^) of DEF1 and DEF2 as determined from fluoride release or dehydrogenase‐coupled assays.

	F^−^ Electrode				Coupled Assay			
Enzyme	DFAA	αFPhAA	αFPA	CFAA	DFAA	αFPhAA	αFPA	CFAA
**DEF1**	**0.12**	**14**	**1.5**	**0.10**	**0.20**	**16.4**	**1.3**	**0.30**
	± 0.00	± 1	± 0.1	± 0.00	± 0.01	± 0.2	± 0.8	± 0.03
**DEF2**	**0.004**	**33**	**0.23**	**0.012**	**0.0026**	**19.6**	**0.74**	**0.0082**
	± 0.000	± 1	± 0.00	± 0.001	± 0.0006	± 1.0	± 0.07	± 0.0002

*Note:* Standard deviations from duplicate (DFAA with the fluoride electrode) or triplicate (all other) assays are shown below the activity values. Electrode values are only measured to the thousandths place.

### 3.5 Testing the Specificity of the Defluorinases With Other Halogens

**FIGURE 4:**
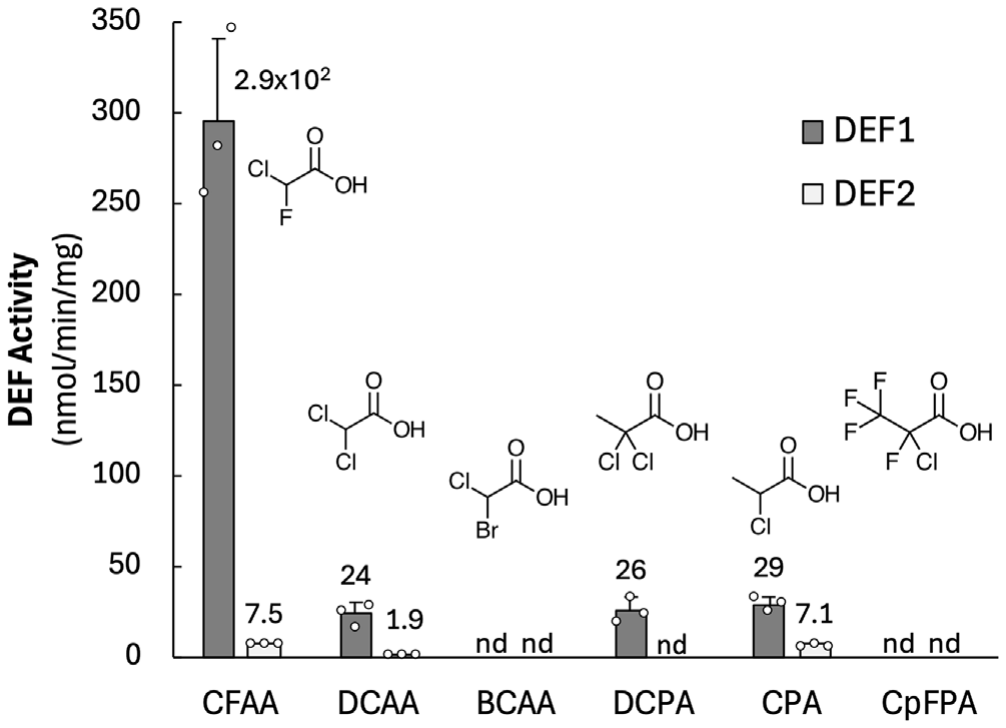
Demonstrating breadth of the coupled assay with multiple halogen substituents, including mixed halogen species. Specific activities of DEF1 ( *Delftia acidovorans* defluorinase) and DEF2 (*Dechloromonas aromatica* defluorinase) with halogenated substrates containing chloro‐ and/or bromogroups using the LDH‐coupled assay. ‘nd’ = not detectable (activity < 1.5 nmol/min/mg).

Our coupled assay theoretically extends to all halides in which displacement produces an α‐hydroxy or α‐ketocarboxylic acid. In this context, we tested a set of mono‐ and *gem*‐dihalo compounds containing chlorine and bromine with DEF1 and DEF2 (Figure 4). CFAA activity is reported in Table 1, but was included here for comparison. Prior to this study, none of the non‐fluoride substrates had been reported except CFAA with DEF1 (Dodge et al. 2024). Both defluorinases were active with α‐chloro‐substituents. The brominated substrate was not active with either enzyme, possibly because the enzyme active site is not large enough to accommodate this larger halogen. Our rate of 295 nmol/min/mg for DEF1 with CFAA is over twofold higher than the value reported by Dodge et al. (2024) of 130 nmol min^−1^ mg^−1^. However, the previous data were reported in Tris at pH 7.5, while ours were at pH 8.0. Our pH study revealed DEF1 is nearly twice as active at pH 8.0 as compared to 7.5. Thus, our values appear to be well aligned.

### 3.6 Using the Coupled Assay to Determine Kinetic Parameters

Any enzyme engineering efforts to improve defluorinase turnover rate, binding affinity or substrate specificity will require basic enzyme kinetics. To demonstrate the applicability of the assay at low defluorination rates, we collected kinetic data using DEF2 and CFAA. DEF2 activity with CFAA is slow (7.5 nmol/min/mg), which is ~4000× lower than DEF2 activity with α‐FPhAA (33,000 nmol/min/mg). Measuring specific activity over a range of CFAA concentrations produced a Michaelis–Menten plot (Figure S5). Data were fitted using Mathematica, and the apparent *K*_m_ and *k*_*cat*_ were determined to be 6.0 mM and 0.32 min^−1^ respectively.

## 4 Discussion

Given the heightened concern about PFAS and other chemicals with halogens *alpha* to a carboxylic acid, monitoring their transformation by microbial enzymes is increasingly of interest. The present study was conducted to assay those transformations more rapidly, in small volumes and via continuous rate curves. The assay was demonstrated for mono‐ and di‐halogenated compounds, and for fluorine‐, chlorine‐, bromine‐ or mixed halogen‐containing carboxylic acids. It was also shown to be applicable for aliphatic or aromatic acids.

The assay described here streamlines the analysis of microbial defluorinating and dechlorinating enzymes that react at the carbon *alpha* to a carboxylate. One previously described alternative method is the use of liquid chromatography‐mass spectrometry (LC–MS) to track substrate disappearance (Sáez et al. 2008). However, extraction of reaction mixtures and determination of the product by LC is discontinuous, labour intensive and requires equipment that is not universally available. Another accepted method for determining dehalogenation enzyme activity is to measure the release of fluoride or chloride. Fluoride determinations can be done using an ion‐specific electrode or via endpoint colourimetric tests (Dhillon et al. 2016; Bygd et al. 2022; Probst et al. 2025). However, fluoride determinations require preparation of the sample to standardise the pH and buffer environment. Generally, greater than one‐half millilitre of liquid is required (depending on the electrode diameter and the sample vessel), and it is a fixed time point assay method. Comparatively, chloride can be determined by fixed time‐point colourimetric assays (West and Coll 1956). Ion chromatography has also been used to determine microbial dehalogenation by comparing peak areas of released halides in comparison to standards (López‐Ruiz 2000). All of these methods suffer from having to sacrifice the sample and determine one time point for each incubation. As such, the methods are time‐consuming and require larger incubation mixtures.

The dehydrogenase‐dependent coupled enzyme assay developed here has several benefits compared to previous methods. First and foremost, coupled enzyme assays give a continuous determination of product formation (Guilbault 1968). As such, continuous assays provide more information about how rates of reaction can change over time. For example, observing rate changes can reveal enzyme activation, inactivation or product inhibition. In general, coupled enzyme assays have a broad utility in biochemical studies (Rudolph et al. 1979). The use of NAD(P) and NAD(P)H is widespread in coupled assay systems due to the significant extinction coefficient of reduced pyridine nucleotides of 6200 M^−1^ and the convenient absorbance maximum of 340 nm (Chenault and Whitesides 1989).

Another utility of the coupled assay described here is that smaller sample sizes can be used. Here, we conducted assays using a standard 1 mL spectrophotometer cell and in microtiter well plates with 200 μL volumes. The latter higher throughput method also provides more opportunity for the determination of steady‐state kinetic parameters that may require a significant number of assay determinations. Compounds containing different halogens may also be studied via the coupled assay as the dehydrogenase reacts with the ketone or alcohol products after halogen displacement.

There are also drawbacks of the dehydrogenase‐dependent coupled enzyme assay with respect to determining defluorination or dechlorination broadly. First, the assay developed here is specifically for carboxylic acid substrates containing one or two halogen substituents on the α‐carbon. This has broad utility with fluoroacetate dehalogenases (Goldman 1965; Chan et al. 2011) and haloacid dehalogenases (Chan et al. 2022; Probst et al. 2025) that evolved to react with fluoroacetate and have activity with other α‐halogenated carboxylate substrates. However, the assay will not be useful for determining defluorination at positions of a fluorinated chain other than at the α‐position. In this context, the current assay does not serve to replace the use of fluoride‐specific electrodes or other methods of halide determination, but it acts as a supplementary tool in the toolbox of assays.

While development and optimisation of a coupled assay was the main focus, it also served to provide new insights on substrate specificity of two defluorinases. The coupled assay revealed previously unknown dehalogenase activity for DEF2 with the substrates chlorofluoroacetic acid, dichloroacetic acid and 2,2‐dichloropropionic acid (Figure 4). Additionally, DEF1 was found to be active with difluoroacetic acid and surprisingly exceeded the known activity of DEF2 by nearly 10‐fold as measured by both fluoride electrode and the coupled assay (Table 1). Moreover, the product of the difluoroacetic acid reactions was confirmed by HPLC to be glyoxylic acid (Figure S4), similar to the previous study with DEF2 (Khusnutdinova et al. 2023). While we did not detect dehalogenase activity with a perhalogenated propionic acid (Figure 4), the lactate dehydrogenase could utilise the proposed 3,3,3‐trifluoropyruvate product, although the activity was low, as shown in Figure 2.

Currently, with the broad interest in PFAS removal from the environment, there are many ongoing efforts to identify new enzymes and improve existing ones via computational and experimental approaches (Husser et al. 2023; Wackett and Robinson 2024; Cunningham et al. 2025; Probst et al. 2025). The enzymes known to hydrolytically displace fluoride from α‐halocarboxylic acids are key targets for re‐engineering their substrate range to include commercial PFAS. The dehydrogenases used in the present study were optimum for the aromatic and short‐chain α‐halocarboxylic acids chosen here. For longer chain PFAS substrates, there is a wealth of microbial NAD‐linked dehydrogenases available that react with larger alcohol and ketone substrates (Reid and Fewson 1994; Hall and Bommarius 2011). The method could be further extended to hydrolytic dehalogenation reactions at multiple positions in a substrate by using NAD‐linked dehydrogenases that catalyse oxidoreduction reactions in complementary positions (Zhou et al. 2020). In this context, the present study pioneers a flexible method for single‐rate determinations, kinetics and high‐throughput screening for a wide range of biodehalogenation reactions.

## Author Contributions

**Marie Ronnander:** methodology, investigation, project administration, visualization, writing – original draft, writing – review and editing. **Anthony G. Dodge:** investigation, resources, writing – review and editing. **Erin O'Neal:** methodology, investigation. **Caroline Pauls:** investigation. **Jack Hanson:** investigation. **James K. Christenson:** project administration, supervision, visualization, writing – original draft, writing – review and editing. **Lawrence P. Wackett:** conceptualization, supervision, project administration, writing – original draft, writing – review and editing, funding acquisition.

## Disclosure

The authors have nothing to report.

## Conflicts of Interest

The authors declare no conflicts of interest.

## Supporting information


**Data S1.** mbt270216‐sup‐0001‐supinfo.pdf

## Data Availability

The data that support the findings of this study are available from the corresponding author upon reasonable request.
