# Supplementary material for: Continuous Spectrophotometric Assay for Defluorinase and Dechlorinase Activities With α‐Halocarboxylic Acids
Source: Microb Biotechnol. 2025 Aug 13;18(8):e70216. doi: 10.1111/1751-7915.70216 (PMC12350541; doi:10.1111/1751-7915.70216)
Supplement: Supplementary file 1 — Data S1. mbt270216‐sup‐0001‐supinfo.pdf [file MBT2-18-e70216-s001.pdf]

Supporting Information

Title: Continuous Spectrophotometric Assay for Defluorinase and Dechlorinase Activities With α-Halocarboxylic Acids

Marie Ronnander[1], Anthony G. Dodge[1], Erin O'Neal[2], Caroline Pauls[2], Jack Hanson[2], James K. Christenson[2*], Lawrence P. Wackett[1*]

Affiliations

1. Department of Biochemistry, Molecular Biology and Biophysics and Biotechnology Institute, University of Minnesota, St. Paul, Minnesota, USA 55108
2. Department of Chemistry, Bethel University, St. Paul, MN, USA 55112

* Corresponding authors:

- James Christenson: chrjam@bethel.edu
- Larry Wackett: wacke003@bethel.edu

Contents

- Table S1 – Table of Strains used in this study
- Figure S1 – Optimized codon sequences for purified dehydrogenases
- Figure S2 – SDS-PAGE of DEFs
- Figure S3 – pH profile of DEFs
- Table S2 – Buffer effects on DEF1 activity with 2,2-difluoracetic acid
- Figure S4 – HPLC trace of DEF1 with 2,2-difluoroacetic acid
- Figure S5 – Kinetics of chlorofluoroacetic acid with DEF2

Table S1. Strains used for the protein purifications in this study.

**Species and strain**	**Genotype or description**	**Reference or source**
P. putida ATCC 12633 + pBBR1-PT5/DEF1	Cloned synthetic fragment containing the gene encoding the *Delftia acidovorans* B defluorinase and a constitutive T5 promoter that drives expression of the enzyme.	Dodge et al. 2024
E. coli BL21(DE3) + pET28b+-DEF2	Inducible expression of the *D. aromatica* RCB defluorinase from a T7 promoter.	O'Connor et al. 2024
E. coli NEB-5α + pET28b+-LDH	Synthetic gene encoding the *L. fermentum* JN248 D-lactate dehydrogenase cloned to be expressed from an inducible T7 promoter.	This study
E. coli BL21(DE3) + pET28b+-LDH	Inducible expression of the *L. fermentum* JN248 D-lactate dehydrogenase from a T7 promoter.	This study
E. coli NEB-5α + pET28b+-MDH	Synthetic gene encoding the *E. faecium* IAM10071 D-mandelate dehydrogenase cloned to be expressed from an inducible T7 promoter.	This study
E. coli BL21(DE3) + pET28b+-MDH	Inducible expression of the *E. faecium* IAM10071 D-mandelate dehydrogenase from an inducible T7 promoter	This study
NEB 5-alpha Competent *E. coli* (High Efficiency)	Cloning strain (*fhuA2Δ(argF-lacZ)U169 phoA glnV44 Φ80Δ(lacZ)M15 gyrA96 recA1 relA1 endA1 thi-1 hsdR17*)	New England Biolabs
E. coli BL21(DE3)	Protein expression strain for T7 promoter (*fhuA2 [lon] ompT gal (λ DE3) [dcm] ΔhsdS; λ DE3 = λ sBamHIo ΔEcoRI-B int::(lacI::PlacUV5::T7 gene1) i21 Δnin5*)	New England Biolabs

>*L. fermentum* D-lactate dehydrogenase gene codon-optimized for *E. coli* expression
ATGGCCAAAATCTATGCATATGGTATTCGCAAAGACGAGGAGCCATATCTTAACGAATGGGCCAAAAATCATGCAGA
TGTGACAGTTGACTATACTGCTGAATTATTGACACCGGAGACTGCCGCACAGGCCGCCGGGGCGGATGGTGTAGTTG
TATACCAGCAATTAGACTATACCGCTGAAACTCTGCAGGCTCTCGCTGACCAGGGCGTTACAAAAATGTCACTCCGC
AATGTAGGGATTGACAATATCGATATGGCAAAGGCCAAGGAACTTGGGTTCGAAATCACGAACGTTCCTGTGTACAG
CCCGAATGCAATCGCGGAACATGCCGCTATTCAGACAGCACGTATCCTTCGTCAATCAAAAAAACTTGATGAGAAGA
TCGAAAATGGTGACCTTCGGTGGGCTCCGACCATCGGGCGTGAGGTTCGTGATCAAGTCGTCGGTGTTGTAGGGACA
GGGCATATTGGGCAGGTATTCATGCAAATTATGGAAGGTTTTGGTGCGAAGGTGATCGCGTACGATGTTTTCAAGGA
CCCTGAACTTGAAAAGAAAGGTTACTACGTCAGTCTGGACGAAATTTACGCGCAAGCCGATGTGATTAGCTTACACG
TTCCGGCACTGGAGTCCACGATTCACATGATTAACGATGAAACTATCGCAAAGATGAAGGACGATGCAGTACTCGTG
AACGTCTCGCGTGGGCCGTTGGTTGATACCGACGCAGTGATCCGCGCTTTGGACAGCGGCAAGTTGTTCGGGTTTGT
CATGGACACATATGAGGACGAAGTCGGGATCTTCAATGAAGACTGGCAGGGGAAAGAGTTCCCAGACGCACGGCTTA
ACGACCTCATCCACCGTGATAATGTTCTTGTCACGCCACATACAGCGTTCTATACGACCCATGCAGTGCGTAACATG
GTCCTGAAAGCGTTCGATAATAATTTGGCCCTCGTGAAAGGCGAAGAGCCTGAAACCCCAGTTAAAGTAGGGTAA

>*E. faecium (1352)* Mandelate dehydrogenase gene codon-optimized for *E. coli* *expression*
ATGAAGATTGCTATCGCCGGTGCCGGTGCCATGGGTTCCCGCTTCGGGTTGATGCTTCACCAGTCGGGCAACGAAGT
TCTTCTGATCGACGGCTGGGCTGAACATGTGCAGCAGATTAAAGAACATGGCCTGCAAGCCAATTTCAACGGGAAGG
AGGTTGAGGCTAAACTTCCAATCGTTTTACAGTCCGAGGTTGAGAAGGAAGATCAAGTCGATCTGATTATTCTTTTT
ACAAAAGCTATGCAGTTGGAGAAGATGTTGCAAGATATTCAGTCCCTCATCAAGAAAGATACCGAGGTCTTATGCCT
CCTGAATGGGATCGGGCATGAGGATATCATTGAGAAGTTCGTGCCGATGGAGAACATCTACATCGGTAATACGATGT
GGACGGCAGGGCTGGAGGGTCCTGGTCAGGTTAAGCTTTTTGGTAGTGGCAGTGTCGAATTGCAGAACCTGGGGGAC
GGCAAAGAAGCTGCAGCGAAAAAATTAGCGGATAAACTTTCTGAATCCGGCCTTAACGCACACTTTAGTGATAACAT
TCATTATTCAATTTATCGTAAAGCGTGCGTAAACGGTACGATGAATGGTCTTTGTACCATTTTAGATGTAAACATGG
CGGAATTGGGCAAGACCTCTACCGCACACAAAATGGTTGCAACTATCGTAAATGAATTCGCAAAAGTCGCCGCCGTT
GAGAAAATCGAGTTGGATGTTCCTGAAGTTATCGCGCACTGTGAAAGTTGTTTTGATCCGGAGACAATTGGCCTCCA
CTACCCATCCATGTACCAAGACTTGATTAAAAACCATCGTCTCACCGAGATCGATTATATCAATGGCGCAATTTCTC
GTAAAGGCAAGAAATACGGTGTAGCCACTCCTTATTGCGACTTCTTGACGGAGCTTGTACACGCTAAAGAAGATTCG
CTGAACGTGAAGTAA

Figure S1. *Protein sequences and codon-optimized gene sequences used for the purification of dehydrogenase enzymes. Optimization was done using Integrated DNA Technologies' codon optimization web tool. Optonized genes included 6x histidine residues and a thrombin cleavage site on the 5' end to facilitate nickle-affinity protein purification after cloning into .*

His-tag affinity purifications of DEF1 and DEF2 proteins

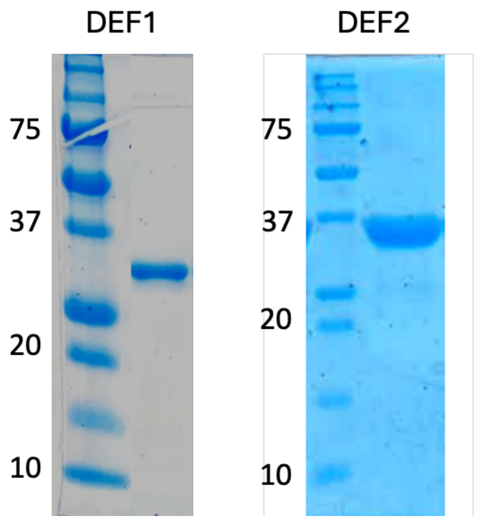

Figure S2. *SDS-PAGE analysis (12.5% gel) to demonstrate the purity of DEF1 and DEF2 purified in a single step by elution from a nickel column with a linear imidazole gradient followed by buffer exchange and concentration. Numbers adjacent to the gel photos are the molecular weights (kDa) of the corresponding standard protein bands.*

DEF1 and DEF2 was most active between 8.0 and 9.0

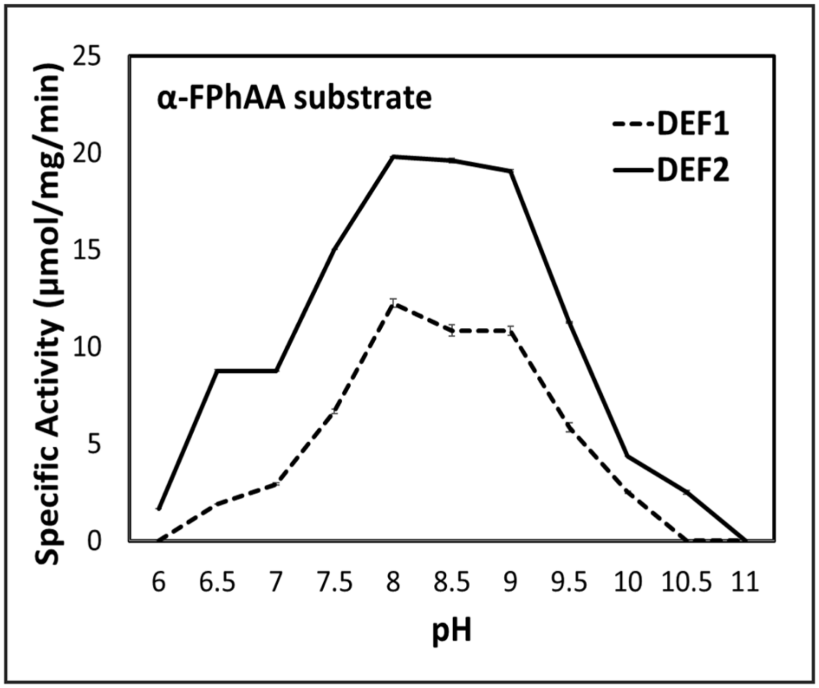

Figure S3. *Plots of specific activity vs pH for DEF2 and DEF2 reacting with 10 mM α-FPhAA.* Activity was determined from measurements taken with a fluoride electrode. Error bars represent the standard deviations of three replicates. Activity was measured from pH 6.0 – 11.0 at increments of 0.5 pH units.

DEF2 is more active with difluoroacetic acid in tris than in other buffers

Table S2. DEF1 activity with difluoroacetic acid

Buffer (0.10M)	pKa	Starting pH	F^- release (20 hr)
Tris	8.1	8.0	46
HEPES	6.8	8.0	13
Phosphate	7.2	8.0	8
Bicine	8.3	8.0	10
Tricine	8.1	8.0	13

HPLC analysis of difluoroacetic acid with DEF1 show glyoxylate as the product

- 1 ml reaction
- 30 mM 2,2-DFAA in 0.1 mM Tris pH 8.5
- 800 µg DEF1 stored in 20 mM HEPES (adds 2.2 mM HEPES)
- ~ 20 h at room temp
- Removed protein w/ 10,000 MWCO centrifugal filter.

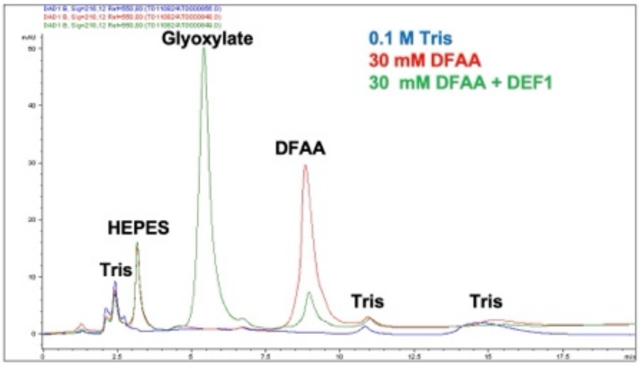

mM F^- released	DFAA peak area – no enzyme added	DFAA peak area + DEF1
50 (83%)	960	175 (18% remained, 82% degraded)

Figure S4. *Overlaid HPLC chromoatgrams of buffer without enzyme or substrate (0.1 M Tris), substrate without enzyme (30 mM DFAA), or substrate + enzyme (30 mM DFAA + DEF1). The glyoxylate peak was identified by comparison to a chromotagram from a glyoxylate commercial standard (not shown). HEPES was added coincidentally into the reaction with DEF1.*

DEF2 exhibits Michaelis Menten kinetics with CFAA

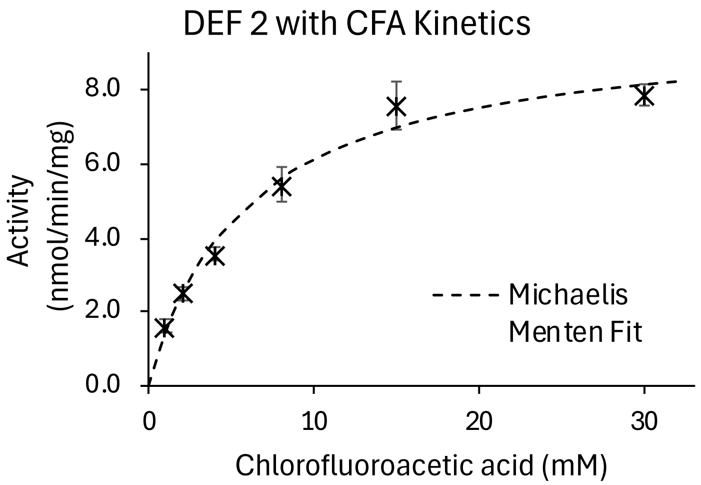

***Figure S5**. Plot of DEF2 specific activity vs [chlorofluoroacetic acid] as determined with the coupled assay to derive kinetic parameters. K_m and k_{cat} were determined to be 6.0 mM and 0.32 min^{-1} respectively.*

References

Dodge, A. G., et al. 2024. "Recombinant *Pseudomonas* Growing on Non-natural Fluorinated Substrates Shows Stress but Overall Tolerance to Cytoplasmically Released Fluoride Anion." *Mbio* **15**: e02785-23.

O'Connor, M. R., C. J. Thoma, A. G. Dodge, and L. P. Wackett. 2024. "Phenotypic Plasticity During Organofluorine Degradation Revealed by Adaptive Evolution." *Microbial Biotechnology* **17**: e70066.
